# Supplementary material for: Mechanisms governing the pioneering and redistribution capabilities of the non-classical pioneer PU.1
Source: Nat Commun. 2020 Jan 21;11:402. doi: 10.1038/s41467-019-13960-2 (PMC6972792; doi:10.1038/s41467-019-13960-2)
Supplement: Supplementary file 7 — Source data [file 41467_2019_13960_MOESM7_ESM.zip › Source_Data/FigureS2/FigureS2B_MotifScanOutput/KmeansCluster5/homerResults/motif6.similar.html]

motif6

## Information for motif6

C
T
G
A
A
T
G
C
C
G
A
T
A
C
G
T
G
T
A
C
A
C
G
T
T
A
C
G
G
A
T
C
C
G
A
T
C
A
G
T
A
G
C
T
A
G
C
T
  
Reverse Opposite:  

T
C
G
A
T
C
G
A
G
T
C
A
G
C
T
A
C
T
A
G
A
T
G
C
C
G
T
A
A
C
T
G
C
G
T
A
C
G
T
A
T
A
C
G
G
A
C
T
  

|  |  |
| --- | --- |
| p-value: | 1e-175 |
| log p-value: | -4.044e+02 |
| Information Content per bp: | 1.678 |
| Number of Target Sequences with motif | 595.0 |
| Percentage of Target Sequences with motif | 14.95% |
| Number of Background Sequences with motif | 1405.7 |
| Percentage of Background Sequences with motif | 3.28% |
| Average Position of motif in Targets | 101.3 +/- 44.3bp |
| Average Position of motif in Background | 97.1 +/- 75.2bp |
| Strand Bias (log2 ratio + to - strand density) | -0.0 |
| Multiplicity (# of sites on avg that occur together) | 1.08 |
| Motif File: | file (matrix) reverse opposite |

### Similar de novo motifs found

|  |  |  |  |  |  |  |  |
| --- | --- | --- | --- | --- | --- | --- | --- |
| Rank | Match Score | Redundant Motif | P-value | log P-value | % of Targets | % of Background | Motif file |
| 1 | 0.947 | C T G A A T G C A C G T A C G T A G T C C G A T T A C G A G T C G C A T G C A T G A C T G A C T G A C T A C G T | 1e-141 | -325.130373 | 7.81% | 0.94% | motif file (matrix) |
| 2 | 0.961 | T G C A C T G A C T G A T C G A C G T A C T A G A G T C C G T A A C T G C G T A C G T A T A C G G A C T | 1e-108 | -250.011008 | 7.39% | 1.21% | motif file (matrix) |
| 3 | 0.783 | C G T A C T A G A G T C C G T A A C T G T C G A C G T A | 1e-76 | -175.684507 | 42.71% | 28.25% | motif file (matrix) |
